# Development of Clean-Label Protein- and Fiber-Enriched Dry Pretzels Using Hemp Seed By-Products

**DOI:** 10.3390/foods14223925

**Published:** 2025-11-17

**Authors:** Tatiana Capcanari, Eugenia Covaliov

**Affiliations:** Department of Food and Nutrition, Technical University of Moldova, 9/9 Studentilor St., MD-2045 Chisinau, Moldova; eugenia.covaliov@toap.utm.md

**Keywords:** hemp, seed cake, pretzels, protein, bioactive profile

## Abstract

This research investigates the incorporation of hemp seed cake (HSC), a nutrient-rich by-product of hemp oil extraction, into traditional pretzel formulations as a sustainable approach to functional dry pretzels development. Wheat flour was substituted with 5–40% HSC, and the resulting products were examined for proximate composition, bioactive profile, and sensory perception. Substitution with hemp seed cake significantly enhanced nutritional quality, raising protein from 7.49 to 12.62% and fiber from 0.90 to 11.78%, while lowering carbohydrates; notably, formulations containing ≥30% HSC meet EU Regulation 1924/2006 criteria to be labeled as a “source of protein.” Functional enrichment was further demonstrated by a four-fold increase in polyphenols (115.28–467.45 mg GAE/kg) and a corresponding rise in total flavonoids (63.40–262.28 mg GAE/kg), as well as by enhanced antioxidant capacity evaluated through DPPH (194.42–658.89 mg Trolox/kg) and ABTS assays (486.05–1647.23 mg Trolox/kg). Sensory analysis (CATA, PCoA) indicated that low substitution levels (≤10%) preserved traditional acceptability, whereas higher levels (≥30%) introduced nutty aroma and denser texture, perceived by consumers as novel. These findings highlight hemp seed cake as a multifunctional ingredient that improves nutritional density, enhances bioactive potential, and supports circular economy principles in the bakery sector.

## 1. Introduction

The oilseed industry is a key contributor to global vegetable oil production; however, it also generates significant agro-industrial waste, presenting major environmental and economic challenges [1]. The by-products generated during oilseed processing, such as oilcakes and meals, are rich in biologically active compounds, valuable nutrients, and high-quality plant proteins [2,3,4]. Despite their potential, inadequate management and utilization strategies result in inefficient use, leading to soil and water pollution, greenhouse gas emissions, and resource wastage [5,6,7]. Globally, more than 2 billion tons of agro-industrial waste are produced annually [1], contributing significantly to environmental degradation and land degradation issues [8,9].

The modern food system faces increasing challenges due to the prevalence of highly processed foods, which often lack essential nutrients. Among the most representative examples are refined bakery products, including white bread and snack-type baked goods (cakes, biscuits, sweet and salted breadsticks), which are globally consumed staple foods but provide predominantly carbohydrates while offering limited protein quality—mainly gluten-forming fractions—and minimal amounts of dietary fiber, vitamins, and minerals due to the extensive refining and processing involved [10]. This dietary shift has contributed to rising health concerns such as obesity, diabetes, and cardiovascular diseases [11,12,13]. The development of enriched bakery products with functional ingredients could significantly enhance dietary quality and improve public health outcomes. Traditional dry pretzels, a staple snack widely consumed in Eastern and Central Europe and increasingly recognized internationally, offer a valuable platform for nutritional enhancement through the incorporation of bioactive compounds and high-quality plant-based ingredients.

Industrial hemp (*Cannabis sativa* L.), an emerging crop with diverse applications and recognized health benefits, presents a valuable but underutilized opportunity for the food industry [14,15,16,17]. Numerous studies have demonstrated that hemp seeds and their derived products contribute to human health through multiple mechanisms. Their balanced ratio of omega-6/omega-3 fatty acids (approximately 3:1) supports cardiovascular health and helps modulate inflammatory responses [18]. Hemp proteins, rich in easily digestible edestin and albumin, supply all essential amino acids and may enhance immune function and muscle metabolism [19]. In addition, hemp seeds are a valuable source of tocopherols, polyphenols, and phytosterols, compounds with documented antioxidant and cholesterol-lowering effects [20]. Recent investigations also highlight the potential neuroprotective and anti-inflammatory roles of hemp bioactives, reinforcing its status as a functional food ingredient [21]. Hemp cultivation produces approximately one ton of seeds per hectare, primarily used for oil extraction; however, the remaining seed cake (approximately 25–30% of total seed mass), commonly referred to as hemp meal, is a protein-rich by-product with excellent nutritional attributes, including a favorable amino acid profile, significant fiber content, and a range of essential minerals [22,23]. According to recent analyses, hemp seed cake contains about 32–35% crude protein, 9–15% lipid fraction, and 30–33% crude fiber, together with 5–6% ash and 0.7% phosphorus [24,25]. The protein fraction comprises all essential amino acids, notably lysine (≈1.1%), methionine (≈0.5%), and arginine (≈4.0%), which are comparable to high-quality animal proteins. In addition, the residual oil contains a balanced proportion of polyunsaturated fatty acids, dominated by linoleic (≈60%) and α-linolenic acids (≈15%), conferring a favorable ω-6/ω-3 ratio [24]. This composition underlines the high nutritional potential of hemp seed cake as a multifunctional ingredient for human food formulations, capable of improving both protein quality and fatty acid profile. Despite these qualities, hemp seed cake remains largely overlooked in human food applications, representing a missed opportunity for nutritional enrichment, waste reduction and sustainable resource utilization.

Recent studies have reported promising results regarding the use of hemp seed cake (HSC) in food matrix formulations, showing improvements in protein, mineral, and polyphenol content, as well as notable changes in dough rheology, color, and sensory perception [22,26,27]. However, existing research has mainly focused on pasta, bread and cookies, whereas traditional dry pretzels remain largely unexplored. Thus, the incorporation of hemp seed cake into traditional products such as pretzels aligns with the principles of functional food development and circular economy practices, offering a viable route to reducing food system waste while addressing nutritional deficiencies.

The aim of this study is to investigate the feasibility of developing nutritionally enhanced traditional dry pretzels through the incorporation of hemp seed cake as a partial substitute for refined wheat flour. This research explores the effects of different inclusion levels of hemp seed cake on the nutritional composition, physical properties, and sensory characteristics of the final product.

## 2. Materials and Methods

### 2.1. Materials

Hemp seed cake, the main functional ingredient used in this study, was sourced from a local oil-producing enterprise in Moldova (producer: “Mira OSF” SRL), where it was obtained by mechanical cold pressing of hemp seeds, a method that typically leaves 8–12% residual oil in the press cake. The accompanying ingredients, including premium-grade wheat flour (10.7% protein according to label, Baneasa producer), refined and deodorized sunflower oil vegetable oil (“Mira OSF” SRL), and salt, were procured from the local Moldovan market. To obtain a uniform flour suitable for bakery applications, the hemp seed cake was first broken into smaller fragments and subsequently ground using a high-speed blender (Panasonic MX-MG5451WTQ, Kadoma, Japan) for 80 s per cycle. The grinding process was repeated as needed until the resulting powder passed entirely through a 250-micron sieve (Endecotts Ltd., London, UK), thereby ensuring consistent particle size distribution and optimal homogeneity when blended with wheat flour.

### 2.2. Methods

#### 2.2.1. Technological Process for Dry Pretzel Preparation

Seven dry pretzel formulations were developed by partially replacing premium wheat flour (type 000) with hemp seed cake (HSC) at varying levels, as shown in Table 1. The substitution levels were set at 0, 5, 10, 15, 20, 30, and 40% (*w*/*w*) of the total flour content. The control sample (P0) contained 100% wheat flour, while the other samples (P5–P40) incorporated progressively higher proportions of HSC. The flour content in each formulation was maintained constant, and the amount of potable water was adjusted accordingly to obtain doughs with similar handling and consistency characteristics. The amount of water was calculated to achieve a dough moisture content of approximately 37%, as recommended by bakery product technology standards. No additional functional additives or dough conditioners were included. All used ingredients were of food-grade quality.

The dry pretzel dough was prepared by mixing the wheat flour and/or HSC-wheat flour mixtures with potable water. Mixing was carried out using a KitchenAid (Artisan^®^ Series 5 Quart Tilt-Head; Greenville, OH, USA) dough mixer (tilt-head stand mixer) for approximately 10 min until a dense, homogeneous, and elastic dough was formed, which could be easily shaped and retained its structure.

After mixing, the dough was allowed to rest at 21 ± 2 °C for 30–60 min. This resting period facilitated the partial development of the gluten network and stabilization of the dough structure. The dough was then divided into portions, rolled into thin cylindrical strands, and manually shaped into rings with a diameter of approximately 2 cm.

The formed rings were subjected to blanching in hot water at a temperature of 85 ± 2 °C for 90 s. This thermal treatment promoted partial gelatinization of the surface starch and led to the formation of a fine, glossy crust, characteristic of traditional dry pretzels. After blanching, the pretzel rings were transferred to baking trays and baked in a baking oven (Fimar, Via Del Tesoro, 301 Villa Veruccihio, Rimini, Italy) at 180 °C for approximately 15 min, ensuring the development of a firm and crisp texture. All pretzel samples were prepared under standardized laboratory conditions using consistent manual techniques simulating artisanal or semi-industrial production. All pretzel samples were packed in sealed polyethylene bags after complete cooling to minimize oxygen exposure and prevent moisture uptake. The samples were stored at 21 ± 2 °C in a dry, dark environment until further analysis. Quality evaluation was performed after 12 h of storage to ensure stabilization of physicochemical and textural properties prior to testing.

#### 2.2.2. Physicochemical Characteristics and Proximate Composition

The proximate composition of hemp seed cake flour (moisture, protein, fat, ash, and crude fiber) was previously determined in our earlier studies [14,22], showing a moisture content of 8.24 ± 0.11%, ash 6.21 ± 0.05% dry matter (d.m.), total protein 31.62 ± 0.22% (d.m.), fat 8.19 ± 0.13% (d.m.), and total dietary fiber 43.76 ± 0.34% (d.m.); therefore, these parameters were not re-evaluated in the present work. In contrast, the proximate composition of the pretzel samples was assessed following standardized procedures, internationally recognized (AOAC).

All analytical determinations were conducted 12 h post-baking, in order to reflect the physicochemical characteristics of the pretzels in conditions closer to those encountered in commercial products. At this stage, the samples were considered stabilized and representative of the final product. Moisture content was measured using the oven drying method in accordance with AOAC Method 925.10, while ash content was determined via incineration following AOAC Method 923.03 [28]. Crude fiber was analyzed according to AOAC 978.10, while crude protein was quantified by the Kjeldahl method AOAC 920.87, which includes digestion, distillation, and titration. Crude fat was determined following the Soxhlet extraction method AOAC 920.85 [28]. Total carbohydrate content was calculated by difference, based on the subtraction of moisture, protein, fat, ash, and fiber from the dry matter [29]. All analyses were performed in triplicate.

Titratable acidity of the pretzel samples was determined following the standardized protocol described in the AACC International Method 02-31.01 [30].

The water activity (a_w_) of the baked samples was determined using a calibrated Rotronic hygrometer (INSTRUMART, Williston, VT, USA), operating on the basis of dew point measurement. Prior to analysis, the device was standardized using certified saline standards to ensure accuracy and reproducibility. Each sample was placed in a sealed measurement chamber under controlled conditions, and the a_w_ value was recorded once equilibrium was achieved [31].

#### 2.2.3. Determination of Total Polyphenol Content (TPC)

The total polyphenol content of the pretzel samples was quantified using the Folin–Ciocalteu spectrophotometric method, as previously described by Makkar et al. (2003) [32]. Briefly, 1 g of finely ground sample was extracted with 10 mL of 70% methanol under constant agitation for 1 h at 21 ± 2 °C. The extracts were then filtered to remove solid residues, and the filtrates were centrifuged. After centrifugation, 0.5 mL of the supernatant was mixed with 2.5 mL of Folin–Ciocalteu reagent (diluted 1:10 with distilled water) and 2.0 mL of 7.5% sodium carbonate (Na_2_CO_3_). The mixture was incubated in the dark for 60 min, and absorbance was measured at 765 nm using a UV–Vis spectrophotometer (Shimadzu UV-1800, Kyoto, Japan). A calibration curve was constructed using gallic acid as a standard, and results were expressed as mg gallic acid equivalents (GAE) per kg of sample [32].

#### 2.2.4. Determination of Total Flavonoid Content (TFC)

The total flavonoid content was assessed according to the procedure reported by Spranger et al. [33], based on the selective precipitation of flavonoid compounds with formaldehyde in a strongly acidic medium. The concentration was obtained from the difference between the total polyphenol content before and after precipitation, and the results were expressed as mg gallic acid equivalents (GAE) per kg of sample.

#### 2.2.5. Determination of Antioxidant Activity (DPPH Assay)

The antioxidant capacity of the samples was evaluated using the DPPH (2,2-diphenyl-1-picrylhydrazyl) radical scavenging assay, following the procedure described by Lin and Zhou (2018) [34], with minor modifications, including adjustment of the DPPH concentration to 0.1 mM, vortex mixing to ensure better homogenization, and reduction in the reaction time to 30 min. A 0.1 mM DPPH solution was prepared in methanol and 2 mL of this solution was mixed with 1 mL of the sample extract. The mixture was vortexed and allowed to react in the dark for 30 min at 21 ± 2 °C. Absorbance was then recorded at 517 nm, and the scavenging activity was quantified by comparing to a Trolox standard curve. Results were expressed as mg Trolox equivalents per kg of sample [34].

#### 2.2.6. Determination of Antioxidant Activity (ABTS Assay)

The antioxidant capacity of the samples was evaluated using the ABTS•^+^ (2,2′-azino-bis (3-ethylbenzothiazoline-6-sulfonic acid)) radical cation decolorization assay, following the procedure described by Arnao et al. [35]. The ABTS•^+^ radical was generated by reacting 7 mM ABTS stock solution with 2.45 mM potassium persulfate, and the mixture was kept in the dark at room temperature for 16 h. The resulting solution was diluted with ethanol to an absorbance of 0.70 ± 0.02 at 734 nm to obtain the working solution. Then, 100 μL of sample extract or Trolox standard was added to 3.9 mL of the ABTS working solution. After 6 min of incubation at 30 °C, the decrease in absorbance was measured at 734 nm. The antioxidant capacity was expressed as mg Trolox equivalents per kg of sample.

#### 2.2.7. Check-All-That-Apply (CATA) Sensory Analysis

The sensory characteristics of the pretzel samples were evaluated using the Check-All-That-Apply (CATA) method [36], 12 h after baking. A panel of 107 untrained consumers (aged 20–55, comprising different genders), recruited from among habitual bakery products consumers, participated in the evaluation. All participants were non-smokers, reported no food allergies, and were instructed to refrain from eating or drinking (except water) for at least 1 h prior to the session. The tests were conducted in the morning (between 9:00 and 11:30 a.m.) in a quiet, ventilated sensory room under daylight-equivalent lighting, with constant temperature (21 ± 2 °C). Each participant received seven coded samples (P0, P5, P10, P15, P20, P30, and P40P0P40, corresponding to 0, 5, 10, 15, 20, 30, and 40% (*w*/*w*) substitution levels of hemp seed cake, presented in randomized and balanced order to minimize bias.

Consumers were instructed to taste each sample and select all terms they considered appropriate from a predefined list of sensory descriptors grouped into eight attribute categories: visual aspect, color, odor, flavor, aftertaste, texture, overall acceptability, and emotional response. The list of terms (Table 2) was developed according to Varela et al. (2012) [37], based on preliminary focus group discussions, product-specific vocabulary, and relevant literature on sensory evaluation of hemp-enriched baked goods, involving a trained panel of 13 assessors who participated in tasting sessions to generate, refine, and agree upon the final set of sensory descriptors [37].

The evaluation took place in a sensory analysis laboratory under controlled conditions (artificial daylight, ambient temperature 21 ± 2 °C, no interfering odors). Water and plain crackers were provided for palate cleansing between samples. Data were collected using paper ballots and subsequently digitized for analysis. The frequency of citation of each term was used for statistical interpretation using Cochran’s Q test and correspondence analysis (CA).

#### 2.2.8. Color Evaluation

The color parameters of the pretzel samples were evaluated using a colorimeter (Konica Minolta CR-400, Tokyo, Japan) operating in the CIELAB color space. The device was calibrated using a white standard tile before each measurement session. For each sample, the color attributes L* (lightness), a* (red-green), and b* (yellow-blue) were recorded in triplicate.

To assess the overall color difference between the control and the fortified samples, the total color difference (ΔE*) was calculated using Equation (1):

(1) $$∆E=\sqrt{{\left(L-L_{0}\right)}^{2}+{\left(a-a_{0}\right)}^{2}+{\left(b-b_{0}\right)}^{2}}$$

where *L*_0_, *a*_0_, and *b*_0_ represent the color values of the control sample.

Additionally, the Whiteness Index (*WI*) was computed to quantify the visual whiteness of the samples, using the following Equation (2):

(2) $$WI=100-\sqrt{{\left(100-L\right)}^{2}+a^{2}+b^{2}}$$

Both Δ*E* and *WI* provide comprehensive insights into the impact of hemp seed cake enrichment on the visual properties of the product surface [38].

#### 2.2.9. Statistical Analysis

All experimental determinations were carried out in triplicate, and the results are presented as mean values accompanied by their respective standard deviations (mean ± SD). A one-way analysis of variance (ANOVA) was applied to assess the statistical significance of differences among samples, using a confidence level of 95% (*p* < 0.05).

In order to explore consumer perceptions, data obtained from the Check-All-That-Apply (CATA) sensory test were processed using multiple analytical approaches. Specifically, Cochran’s Q test was used to determine whether significant associations existed between the evaluated samples and the sensory attributes identified by the panelists [37].

Pearson correlation analysis was conducted to elucidate the interrelationships among key technological, nutritional, and functional parameters of hemp-fortified pretzels. This multivariate statistical approach facilitated the identification of significant positive or negative associations between variables such as hemp seed cake concentration, moisture, protein, acidity, water activity, fiber, carbohydrate total polyphenol content, antioxidant activity, and whiteness index. The resulting correlation matrix was visualized as a heatmap (Figure 3), offering mechanistic insights into the patterns observed across the experimental formulations [39].

All statistical analyses were performed using XLStat software (version 7.5.2, Addinsoft, Paris, France) integrated into Microsoft Excel. One-way ANOVA was applied to identify significant differences among samples, followed by Tukey’s HSD post hoc test (*p* < 0.05) for mean comparison.

## 3. Results

### 3.1. Physicochemical Characteristics of Pretzels

In order to evaluate the technological feasibility and functional impact of hemp seed cake incorporation into pretzel formulations, a comprehensive analysis was conducted on moisture, acidity, water activity, macronutrient content, bioactive compounds, and consumer perception. Table 3 presents the key physicochemical characteristics of pretzels produced by gradually replacing refined wheat flour with hemp seed cake at levels from 0 to 40%. The evaluated parameters—moisture content, titratable acidity and water activity (a_w_)—were selected to capture the technological behavior and functional enhancement introduced by HSC.

Among the evaluated parameters, moisture content was considered first, as it directly influences textural properties, microbial stability, and regulatory compliance in bakery products, and provides a baseline for assessing the technological impact of hemp seed cake incorporation. The moisture content of the pretzels slightly decreased from 12.48 in the control sample (P0) to 11.36% in the 40% HSC formulation (P40). All values complied with the national regulatory standard of the Republic of Moldova, which sets a maximum permissible moisture content of 13% for such type of bakery products [40]. Similar decreasing trends in moisture with increased substitution of protein- and fiber-rich ingredients have been reported in hemp-fortified gluten free bread [41] and legume-based bakery products [42].

The titratable acidity increased progressively with the level of HSC, from 1.67 degrees in the control to 2.71 degrees in P40. This rise can be attributed to the presence of organic acids and polyphenols in hemp cake, which contribute to a slightly tangy or earthy sensory profile. According to Menga et al. (2022) [43], the main phenolic compounds present in hemp seed products include N-trans-caffeoyltyramine, p-coumaric acid, ferulic acid, caffeic acid, vanillic acid, syringic acid, and p-hydroxybenzoic acid, along with flavonoids such as epicatechin and naringenin, all of which are associated with antioxidant and mildly acidic characteristics [43]. Despite the progressive increase, all obtained samples complied with the applicable national regulatory framework, as the maximum permissible acidity for this type bakery products is 3 degrees according to Decision of the Government of the Republic of Moldova No. 775 from 2007 on the approval of the Technical Regulation “Bakery products and pasta” [40]. Comparable increasing trends in acidity were observed by Rusu et al. (2021) in hemp-fortified bakery products, where residual lipids and phenolic compounds contributed to higher titratable acidity [26]. Bădărău et al. (2018) similarly noted that inclusion of hemp flour significantly elevated acidity in bread [44].

Water activity (a_w_) was evaluated as a predictor of microbial stability and shelf-life extension. Lower a_w_ values indicate less free water, which can reduce spoilage risk in dry bakery products. Water activity decreased as the substitution of wheat flour with HSC increased, ranging from 0.524 (P0) to 0.428 (P40). This reduction in a_w_ is primarily due to the high fiberfiber and protein content of hemp cake, which binds water molecules, reducing free water in the system. Similar reductions in a_w_ were observed in corn-hemp flour composites and high-fiber bakery formulations, which improve microbial stability due to limited water availability [45]. Ogunronbi et al. (2011) also confirmed that oilseed press cake incorporation in bakery matrices lowers a_w_ and enhances storage stability [46]. In agreement, Alp and Bulantekin (2021) reported that reduced water activity achieved through drying or ingredient modification effectively inhibits microbial proliferation and extends the microbiological safety and shelf-life of food products [47].

### 3.2. Proximate Composition of Pretzels

The proximate composition analysis presented in Table 4 demonstrates a gradual and statistically significant increase (*p* < 0.05) in protein, fiber, ash, and fat content with higher substitution levels of hemp seed cake, while carbohydrate content tended to decrease accordingly.

Crude protein content showed a proportional rise, from 7.49 (P0) to 12.62% (P40), with each substitution level showing statistically significant differences. This is consistent with the high protein concentration of hemp cake, which contains all essential amino acids. The amino acid profile of hemp seed products, confirming the presence of all essential amino acids, was demonstrated in our previous study [22]. Comparable results were reported by Wiedemair et al. (2022), who incorporated hemp press cake and grits into bread formulations, achieving increases in protein content from 12 to 54% [48]. According to Regulation (EC) No 1924/2006, a food product can be labeled as a “source of protein” if proteins contribute at least 12% of its total energy value, and as “high in protein” if the contribution reaches or exceeds 20% [49]. In this study, the energy contribution of protein was calculated based on the Atwater conversion factor (4 kcal/g protein), confirming that the P30 (13.82%) and P40 (15.81%) samples meet the threshold for a “source of protein” claim. This finding is relevant for the development of clean-label bakery products with functional and health-promoting potential, especially for consumers interested in plant-based protein enrichment.

Pretzel fat content ranged from 3.13 to 4.98%, remaining within the acceptable range of 3–15% defined by national technical regulations [40]. The moderate increase in lipid levels is likely due to residual oil retained in the partially defatted hemp cake. Similar tendencies for fat content were obtained in studies using pumpkin seed press cake flour in crackers [50]. Crude fiber increased markedly from 0.90 to 11.78% across the substitution gradient, confirming the fiber-rich nature of hemp residues. This represents a significant improvement in the functional quality of the product, aligning with trends in clean-label, high-fiber bakery innovations. Comparable improvements were reported by Makowska et al. (2023), who used flax cake and lupine flour in bread, increasing fiber from 2.6 to over 13.76% [51]. The ash content increased significantly (*p* < 0.05) from 1.79 in the control to 3.27% in P40, indicating an enhanced mineral profile. These values are comparable to those reported by Korus et al. (2017), who observed ash contents between 8.7 and 149.2 g∙kg^−1^ in wheat biscuits enriched with 20–60% hemp flour [45]. From previous studies in clear that the addition of hemp seed cake increases the content of Mg, K, P Mn, Fe, Zn and Cu [22].

Inversely, carbohydrate content decreased from 74.20 in the control sample to 55.99% in the P40, reflecting the dilution of starch-rich wheat flour with fiber- and protein-dense material. Such reductions in carbohydrate proportion have also been reported by Martínez et al. (2025) when enriching baked goods with nut-based press cakes [52].

### 3.3. Total Polyphenol, Flavonoid and Antioxidant Activity of Pretzels

The incorporation of hemp seed cake substantially enhanced the functional profile of the pretzels, as reflected by total polyphenol content (TPC) and antioxidant activity (Table 5). The TPC increased progressively from 115.28 mg GAE/kg in the control (P0) to 467.45 mg GAE/kg in P40, indicating a strong enrichment with phenolic acids, flavonoids, and lignanamides naturally present in hemp by-products. In a related study, Pecyna et al. (2023) [53] observed that fortifying gluten-free bread with 1–5% hemp inflorescences increased the total polyphenol content up to approximately 400 mg GAE/kg, accompanied by enhanced antioxidant activity. This confirms that even low to moderate levels of hemp enrichment can significantly improve the functional quality of bakery products [53]. Similarly, Ambroziak et al. (2025) reported that hemp seed cakes—particularly those obtained from dehulled seeds—retain substantial amounts of phenolic compounds and exhibit strong oxidative stability, maintaining polyphenol levels in the range of 350–450 mg GAE/kg depending on the formulation [54]. Comparable observations were reported for bakery products enriched with nut-based press cakes, which similarly enhance polyphenolic content and functional properties [52].

The determination of total flavonoid content (TFC) provided further insight into the bioactive profile of the enriched pretzels. TFC values ranged from 63.40 mg GAE/kg in the control sample to 262.28 mg GAE/kg in P40, showing a strong positive correlation (r ≈ 0.96) with the polyphenolic fraction. This suggests that flavonoids represent a major contributing group to the overall antioxidant potential of hemp-fortified formulations. In agreement with our findings, Benkirane et al. (2022) [55] highlighted that hemp seed extracts are rich in phenolic compounds, particularly flavonoids, which represent major contributors to the total phenolic fraction and the radical scavenging activity, emphasizing their key role in the antioxidant potential of hemp-derived products [55].

This enrichment in polyphenols translated into a marked increase in antioxidant capacity, evaluated by DPPH radical scavenging activity. The pretzels exhibited a clear upward trend from 194.42 in the control (P0) to 658.89 mg Trolox/kg in P40, reflecting the contribution of polyphenols and other bioactive compounds inherent to hemp cake. Martínez et al. (2025) similarly demonstrated that bakery products enriched with nut-based press cakes exhibited significantly enhanced antioxidant potential due to the synergy of retained polyphenols and Maillard reaction products formed during baking [52]. Menga et al. (2022) further highlighted that hemp derivatives contribute not only to radical scavenging activity but also to the development of health-oriented functional foods enriched in plant antioxidants [43].

Moreover, the antioxidant activity determined by the ABTS•^+^ method followed the same dose-dependent pattern, with values increasing from 486.05 (P0) to 1 647.23 mg Trolox/kg (P40). This confirms the consistency between DPPH and ABTS assays and indicates that the antioxidant response of hemp-fortified pretzels is multifaceted, involving both hydrophilic and lipophilic radical scavenging mechanisms. The strong linear relationship between ABTS values and total polyphenol/flavonoid content suggests that the majority of the antioxidant activity originates from phenolic compounds, particularly flavonoids and lignanamides. The progressive increase in TPC, TFC and antioxidant activity confirms that hemp seed cake acts as a potent functional ingredient, enhancing the nutritional and bioactive profile of pretzels while aligning with current trends in clean-label, health-promoting bakery products.

### 3.4. Sensory Evaluation of Pretzels

The sensory evaluation of pretzels with increasing levels of hemp seed cake was supported by visual inspection of dough and baked samples across formulations (Table 6) and advanced consumer profiling using CATA data, further explored through Correspondence Analysis (CA).

#### 3.4.1. Sensorial Data Analysis Based on CATA Results

The sensory profiling of the pretzel samples enriched with varying levels of hemp seed cake (0–40% *w*/*w*) was conducted using the Check-All-That-Apply (CATA) method. Consumer responses were recorded as a binary data matrix reflecting the frequency of selection for each predefined sensory attribute. To identify terms that effectively discriminated between samples, the data were subjected to Cochran’s Q test. From the original set of 53 descriptors included in the CATA questionnaire, 22 showed statistically significant differences (*p* < 0.05) and were subsequently retained for multivariate analysis. These selected attributes were further explored using Correspondence Analysis (CA) and Principal Coordinate Analysis (PCoA) to visualize both the sensory positioning of the samples and the structure of perceptual relationships among attributes.

**Figure 1:**
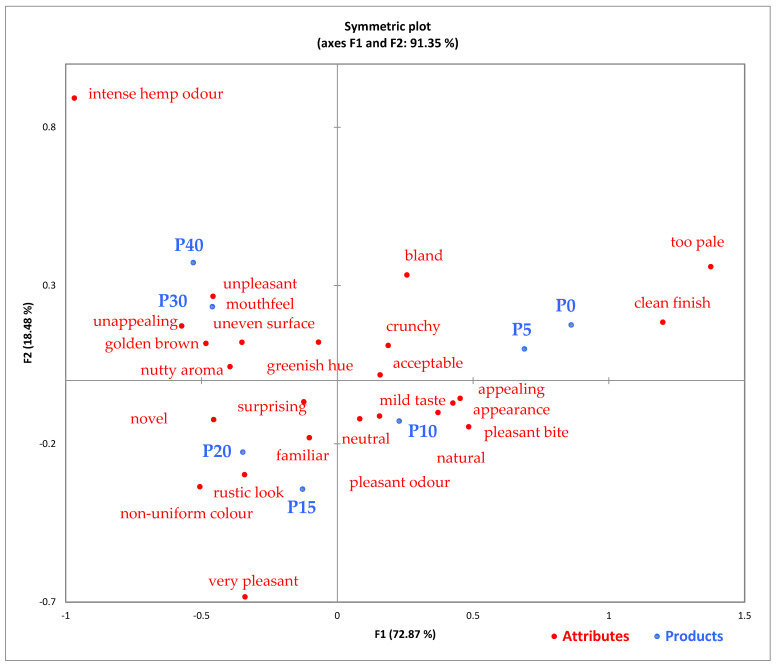
Correspondence Analysis (CA) biplot of pretzel samples and CATA attributes. P0—control sample, P5—pretzel with 5% addition of hempseed cake, P10—pretzel with 10% addition of hempseed cake, P15—pretzel with 15% addition of hempseed cake, P20—pretzel with 20% addition of hempseed cake, P30—pretzel with 30% addition of hempseed cake, P40—pretzel with 40% addition of hempseed cake.

The CA biplot (Figure 1) accounted for 91.35% of the total variation across the first two axes, with Dimension 1 (F1) explaining 72.87% and Dimension 2 (F2) 18.48%. This representation clearly discriminated the pretzel samples based on their sensory profiles. While F1 primarily reflected the effect of hemp seed cake substitution level, separating the control and low-substitution samples from the high-substitution ones, F2 captured the intensity and character of sensory perception by distinguishing milder and familiar notes from stronger hemp-related sensations.

The control sample P0 and the low substitution variant P5 clustered on the right side of the biplot and were strongly associated with favorable attributes such as pleasant bite, appealing appearance, clean finish, golden brown, and pleasant odor, suggesting high acceptability and classical sensory characteristics. These sensory advantages are explained by the predominance of gluten-forming proteins and the absence of hemp pigments, which ensured a well-developed structure and a light golden coloration typical of conventional pretzels. Conversely, along the vertical axis (F2), samples positioned higher in the plot (P30–P40) exhibited intense hemp odor, uneven surface, and unappealing appearance, reflecting stronger and less desirable attributes, whereas those positioned lower (P15–P20) were associated with mild taste, familiar and rustic appearance, and pleasant odor. This indicates that F2 represents the transition from pleasant and familiar to intense and distinctive hemp-related sensory characteristics. Similar trends were observed by Korus et al. (2017) in hemp flour-enriched cookies, where low substitution levels preserved traditional sensory traits and consumer acceptance [41]. P10 remained close to the neutral and mild descriptors, indicating minimal sensory deviation. At this substitution level, the limited inclusion of hemp cake did not significantly alter the dough rheology or flavor profile, maintaining an optimal balance between texture and aroma. This is consistent with findings by Sadohara et al. (2022), who showed that moderate incorporation of pulse-based flours into baked products minimally affects overall liking [56].

Samples P15 and P20 occupied an intermediate space in the plot, associated with more complex descriptors such as natural, rustic look, familiar, and surprising, reflecting the onset of perceptible changes while maintaining moderate consumer acceptance. These tendencies can be attributed to partial dilution of the gluten network and the increased presence of dietary fiber, which slightly reduced dough elasticity and contributed to a denser internal structure. In addition, the intensification of earthy and nutty notes results from the higher content of phenolic compounds and residual lipids specific to hemp seed cake. Similar descriptors were found to emerge in breads enriched with functional plant ingredients, as reported by Dziki et al. (2014) and by Samilyk et al. (2025), where increasing substitution led to a shift in perception toward natural and rustic qualities [57,58].

Samples P30 and P40 were located in the upper left quadrant, near terms such as greenish hue, nutty aroma, and unpleasant mouthfeel, suggesting noticeable shifts in visual and textural properties. The pronounced darkening and greenish tones correspond to the accumulation of chlorophyll and Maillard browning pigments, while the compact structure is a direct consequence of reduced gluten development and higher fiber concentration. These compositional effects also explain the stronger hemp-like aroma and slightly bitter aftertaste perceived by the panelists. Comparable findings were reported by Mikulec et al. (2019), Rusu et al. (2021), where breads enriched with high levels of hemp cake developed darker colors and intensified nutty and earthy flavors, some perceived as overpowering by untrained consumers [26,59].

Importantly, the attribute novel was also strongly associated with P20 and P30, indicating that these formulations were perceived by consumers as original and different from conventional products. Comparable interpretation of this term was made by Lazou (2023) in a study on legume-based biscuits, where higher substitution was linked to a significant increase in perceived novelty and innovativeness, despite textural compromises [60]. This pattern suggests that, while higher hemp inclusion introduces sensory deviations, it simultaneously enhances the perception of authenticity and product innovation, a valuable feature for functional or health-oriented bakery formulations.

#### 3.4.2. Sensorial Data Analysis Based on Principal Coordinate Analysis (PCoA)

**Figure 2:**
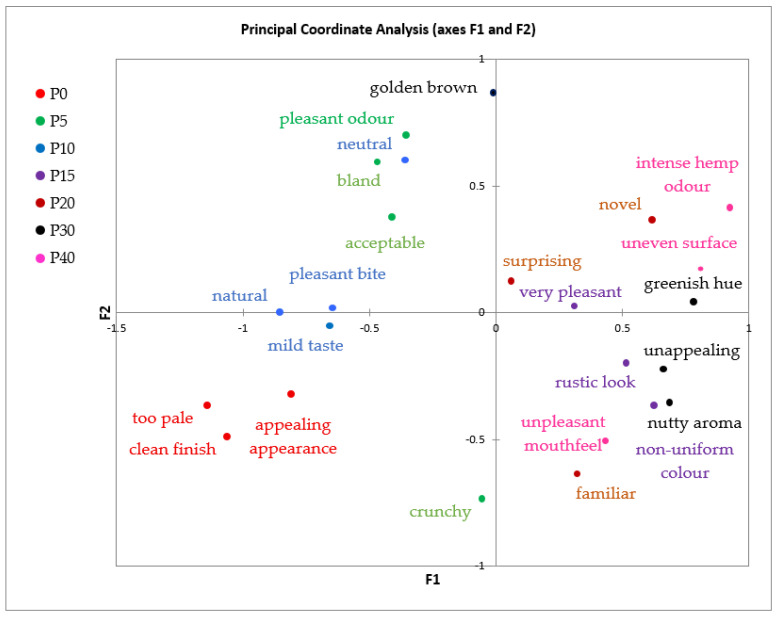
Principal Coordinate Analysis (PCoA) of sensory attributes derived from CATA data. P0—control sample, P5—pretzel with 5% addition of hempseed cake, P10—pretzel with 10% addition of hempseed cake, P15—pretzel with 15% addition of hempseed cake, P20—pretzel with 20% addition of hempseed cake, P30—pretzel with 30% addition of hempseed cake, P40—pretzel with 40% addition of hempseed cake.

To further explore the internal structure of sensory attributes and reduce potential dependence on sample coding, Principal Coordinate Analysis was performed using the same CATA data (Figure 2). The PCoA plot provided an unsupervised view of the similarity among sensory descriptors. Although the cumulative variance explained is not directly specified, the dispersion suggests a meaningful two-dimensional representation.

PCoA confirmed the CA findings by grouping attributes associated with control and low-substitution samples (e.g., pleasant bite, natural, neutral, pleasant odor, mild taste) on the left side of the graph, while descriptors such as greenish hue, unappealing, and uneven surface appeared toward the right. Comparable clustering patterns were reported by Cunha et al. (2019), who employed PCoA on CATA data from legume-enriched bakery items and noted that traditional descriptors like “crisp” and “familiar” clustered close to low-substitution variants [61].

The attribute “novel” was positioned in the upper right quadrant of the PCoA map, in close proximity to greenish hue and nutty aroma. This positioning reinforces its role as a perceptual marker of originality and functional reformulation. Rather than being perceived negatively, “novel” expresses consumer recognition of novel sensory profiles specific to high hemp content. Similar associations between “novel” and descriptors such as “earthy” or “uncommon taste” were documented in studies evaluating consumer responses to functional bread enriched with insect flour or plant-based proteins [62,63]. In this context, “novel” bridges the sensory transition between conventional expectations and the development of value-added bakery products.

The vertical axis (F2) differentiated attributes based on intensity and visual complexity, while the horizontal axis (F1) separated familiar and mild descriptors from bold and distinctive sensory cues. These dimensional interpretations align with findings by Yang (2025), who demonstrated that in PCoA of CATA data, F1 commonly reflects familiarity or hedonic tone, whereas F2 reveals intensity or cognitive activation [64].

Both CA and PCoA confirmed that hemp seed cake can be integrated in pretzel formulations across a wide substitution range, with different sensory outcomes. Up to 15–20% substitution supports traditional acceptability with minimal sensory disruption. Beyond this level, distinctive characteristics emerge—greenish hue, nutty aroma, and dense texture—accompanied by positive perceptions of “novelty”, suggesting interest and acceptance of product differentiation. Comparable conclusions were reported by Jarecki et al. (2024) in their study on bakery products with amaranth, flax and hemp seeds, where high substitution levels modified the appearance and mouthfeel but were still positively accepted by consumers due to health and novelty associations [65].

The consistent appearance of “novel” in samples P30 and P40 underscores their potential as functional alternatives to conventional bakery items, targeting consumers who seek novelty, health attributes, and sustainable ingredients. Rather than being a marker of rejection, “novel” reflects a shift toward exploratory sensory appreciation. This reflects the consumer segmentation described by Tuorila et al. (2020), where “innovator” consumers positively associate non-traditional attributes with health benefits and originality [66]. The multivariate analyses thus support a dual positioning strategy for hemp-enriched pretzels—maintaining classical formulations for broad consumer bases, while promoting higher-substitution variants as innovative and functionally enriched products suitable for niche and health-conscious markets.

### 3.5. Color Parameters

Color is one of the most immediate and influential sensory attributes affecting consumer perception and acceptability of bakery products. The CIELAB parameters (L*, a*, b*) were determined for the pretzel dough and the final baked samples to evaluate the impact of hempseed cake addition at various substitution levels (5–40%).

As shown in Table 7, the lightness parameter (L*) decreased significantly (*p* ≤ 0.05) with increasing levels of hempseed cake. For dough, L* values decreased from 97.40 (P0) to 54.21 (P40), while for final products, L* dropped from 82.39 to 27.15. This pronounced darkening effect reflects the color of the hempseed cake and its influence on the visual appearance of the matrix.

The a* values (red–green axis) showed a progressive increase from negative values in the control dough (−1.98), indicating a greenish hue, to slightly positive values up to 3.56 for P15, suggesting a gradual shift toward red tones in the unbaked samples. This sudden increase in a* values at 15% substitution reflects the cumulative effect of pigments and phenolic compounds present in the hemp seed cake, which contribute to brown–reddish shades even before baking. Phenolic acids such as ferulic and *p*-coumaric acid, abundant in hemp meal, are known to undergo partial oxidation during dough mixing and resting, producing colored quinones that intensify warm hues. In the final baked pretzels, this trend was more pronounced, with a* values reaching 8.99 for P30, likely due to intensified Maillard and caramelization reactions between reducing sugars and amino compounds during baking, leading to the formation of melanoidins responsible for reddish–brown coloration. Additionally, the presence of polyunsaturated fatty acids and residual chlorophyll derivatives may interact with thermal degradation products, further deepening color intensity. This is in agreement with observations by Lewandowicz et al. (2025), who reported a similar increase in a* values in bakery products fortified with hemp and legume proteins due to the formation of melanoidins and other browned pigments [67].

The b* values (yellow–blue axis) exhibited variable behavior with increasing HSC substitution rather than a consistent decrease. Moderate substitution levels (P5–P20) showed slightly elevated b* values, corresponding to a more intense yellow tone, while higher levels of hemp seed cake (≥30%) resulted in darker, duller hues. Comparable variations in b* were observed by Nemś et al. (2022) in cookies enriched with plant-based press cakes, particularly those high in polyphenols and chlorophyll-like compounds [68]. These fluctuations may be explained by the combined influence of natural carotenoids present in wheat flour, early-stage Maillard reaction intermediates formed during baking, and the masking effect of chlorophyll and phenolic pigments introduced by the hemp seed cake [69,70].

The total color difference (Δ*E*) compared to the control (P0) confirmed perceptible changes across all samples. In dough, Δ*E* ranged from 9.73 (P5) to 45.43 (P40), while in baked pretzels it reached 55.62 at 40% HSC. These values are well above the just noticeable difference (JND) threshold of approximately 2.3 in the CIELAB color space. Δ*E* values around 2.3 have been defined as the JND for human observers according to the CIE76 standard [71]. Additionally, De Flaviis and Sacchetti (2025) confirmed perceptibility thresholds of Δ*E* = 1.25 for L* and a* and Δ*E* = 2.8 for b*, further underpinning the assertion that Δ*E* values in our study are distinctly perceptible by untrained observers [72]. Similarly, Levent et al. (2020) reported Δ*E* values above 40 in cereal products fortified with grape and seed press cakes, highlighting the strong pigmenting effect of plant-based residues [73].

The Whiteness Index (*WI*) decreased significantly (*p* ≤ 0.05) with HSC addition, from 95.01 (P0) to 44.32 (P40) in dough, and from 89.22 to 36.86 in pretzels. This darkening effect is attributed to the pigment-rich nature of hemp ingredients, rich in chlorophyll and phenolics. The visual darkening observed through WI measurements was consistent with sensory outcomes, as confirmed by the CATA (Figure 1) and PCoA (Figure 2) analyses. Samples with lower WI values (P30–P40) were associated with descriptors such as “unappealing,” “uneven surface,” and “intense hemp odor,” indicating a direct link between color loss and decreased visual acceptability. Conversely, samples with higher WI values (P0–P10) clustered with favorable attributes including “pleasant bite,” “appealing appearance,” and “clean finish.” This alignment between instrumental and sensory data confirms that the reduction in WI not only reflects pigment incorporation but also perceptibly affects consumer perception of product attractiveness. Similar trends were reported by Korus et al. (2017), who found decreased *WI* in biscuits fortified with 20–60% hemp flour [45].

### 3.6. Interrelationships Between Hemp Seed Cake Addition and Pretzel Quality Attributes

**Figure 3:**
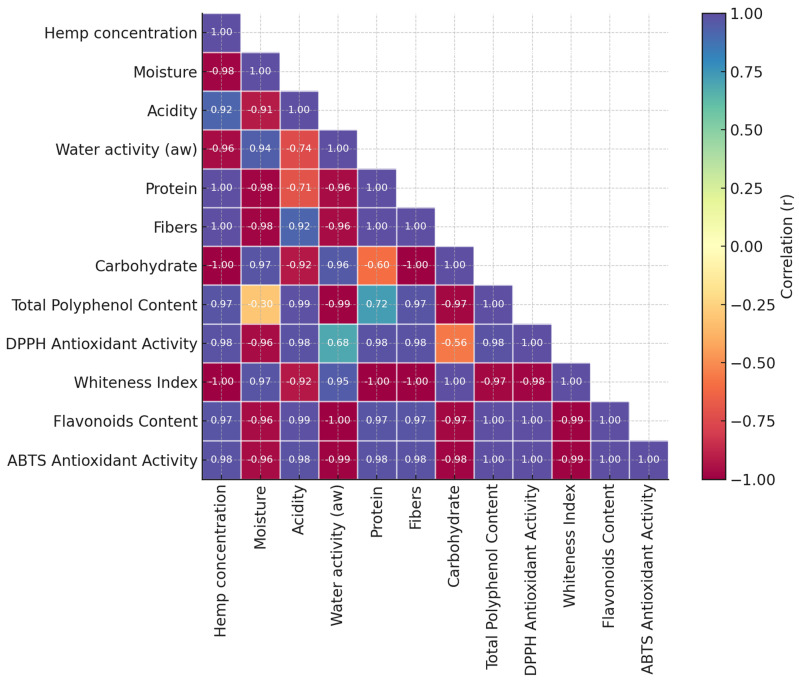
Pearson correlation heatmap between technological, nutritional, and functional parameters of hemp-fortified pretzels.

Pearson correlation analysis (Figure 3) was performed to elucidate the interrelationships between hemp seed cake addition and the key technological and nutritional parameters of fortified pretzels.

A strong negative correlation was observed between protein and carbohydrate content (*r* = −0.60), highlighting the macronutrient shift induced by HSC addition. This trend aligns with the proximate composition results, where protein enrichment occurred at the expense of carbohydrates. From a nutritional perspective, this shift contributes to improved protein density and lower glycemic potential, enhancing the functional value of the final product. Technologically, the reduction in carbohydrate content and gluten-forming fractions, together with the higher level of hemp proteins, may alter dough rheology and baking performance, influencing texture and color development. Similarly, protein showed a significant inverse correlation with acidity (*r* = −0.71), possibly due to the dilution or buffering effect of the protein-rich matrix [74]. Conversely, protein and total polyphenol content were positively associated (*r* = 0.72), reflecting the co-extraction of phenolic compounds along with proteins from hempseed press cake [75]. This association is consistent with the observed increase in antioxidant capacity and phenolic content in higher substitution levels. Total flavonoid content was strongly and positively correlated with total polyphenol content (r ≈ 0.99) and antioxidant activities (DPPH and ABTS; r > 0.98), confirming that flavonoids represent a major fraction of the antioxidant system in hemp-enriched pretzels. From a mechanistic perspective, flavonoids contribute both through hydrogen-donating capacity and metal-ion chelation, which stabilize free radicals and prevent oxidative damage. Their strong co-variation with total polyphenols and antioxidant indices suggests a synergistic contribution of multiple phenolic subclasses, reinforcing the overall biofunctional potential of the fortified formulations.

Notably, antioxidant activity (DPPH) was moderately correlated with water activity (*r* = 0.68), indicating a possible synergistic effect between moisture and the availability of active compounds. This relationship may be explained by the role of bound and unbound water in modulating the solubility and diffusivity of phenolic compounds. A moderate increase in water activity can enhance molecular mobility [76] and facilitate radical scavenging reactions, thereby improving measurable antioxidant activity. However, excessive moisture may also accelerate oxidative reactions or degradation of sensitive bioactive elements, suggesting an optimal hydration range for maintaining antioxidant potential [77]. At the same time, DPPH and ABTS antioxidant activity exhibited a clear negative correlation with carbohydrate content (*r* = −0.56 and *r* = −0.98), suggesting that samples richer in reducing sugars may exhibit faster oxidative degradation. Moisture was inversely related to total polyphenol content (*r* = −0.30), which could reflect water-induced dilution effects or matrix interactions [78]. Furthermore, a high inverse correlation was found between acidity and water activity (*r* = −0.74), potentially linked to shifts in free water availability during acidification.

## 4. Conclusions

The incorporation of hemp seed cake into dry pretzel formulations provided measurable nutritional and functional improvements while maintaining product feasibility. At 30–40% substitution, protein content increased by more than 65% (from 7.49 to 12.62%), fiber content rose over ten-fold (0.90–11.78%), and polyphenols and antioxidant activity reached 467.45 mg GAE/kg and 658.89 mg Trolox/kg, respectively. The total flavonoid content increased more than four-fold (63.40–262.28 mg GAE/kg), confirming the enrichment of the bioactive matrix with flavonoid-type compounds. Antioxidant activity assessed by the ABTS assay (486.05–1647.23 mg Trolox/kg) showed the same dose-dependent trend as DPPH, demonstrating the consistency of results across methods and indicating a broader radical scavenging spectrum. These values confirm the potential of dry pretzels to be marketed as a “source of protein” according to EU Regulation 1924/2006. Nevertheless, further optimization is needed to improve sensory acceptability at higher substitution levels. Sensory evaluation revealed that up to 20% hemp seed cake ensured broad acceptability, while higher levels were associated with distinctive attributes-nutty aroma, dense texture, and a perception of novelty-appealing to niche consumer groups. Correlation analysis reinforced these outcomes, showing a strong positive link between protein and polyphenols (*r* = 0.72) and a negative correlation between carbohydrates and antioxidant activity (*r* = −0.56). The study demonstrates that hemp seed cake can transform conventional dry pretzels into functional products with enhanced bioactive potential, while valorizing an agro-industrial by-product within a circular economy framework.

## Figures and Tables

**Table 1:** Pretzel formulations.

Raw Materials, g	Hemp Seed Cake Level, % (% Replacement of Wheat Flour)						
	0%	5%	10%	15%	20%	30%	40%
Wheat flour	70.0	66.5	63.0	59.5	56.0	49.0	42.0
Hemp seed cake	0.0	3.5	7.0	10.5	14.0	21.0	28.0
Vegetable oil	2.5	2.5	2.5	2.5	2.5	2.5	2.5
Salt	1.5	1.5	1.5	1.5	1.5	1.5	1.5

**Table 2:** CATA attributes for pretzel sensory analysis.

Attribute Category	CATA Attributes
Visual Aspect	Appealing appearance, uneven surface, rough crust, cracks on surface, attractive shape, rustic look
Color	Too pale, golden brown, dark brown, greenish hue, uniform color, non-uniform color
Odor	Intense hemp odor, weak hemp odor, nutty aroma, earthy odor, roasted smell, floury smell, pleasant odor
Flavor	Mild taste, bitter, nutty, fermented, salty, savoury, bland, unpleasant taste
Aftertaste	Lingering bitterness, nutty aftertaste, clean finish, herbal aftertaste, unpleasant residual taste
Texture	Hard, crunchy, crumbly, dry, gritty, compact, dense, pleasant bite, unpleasant mouthfeel
Overall Acceptability	Very pleasant, acceptable, neutral, slightly unpleasant, unpleasant
Emotional Response	Comforting, familiar, novel, surprising, strange, natural, unappealing

**Table 3:** Quality parameters of pretzels enriched with hemp seed cake.

Pretzels Sample	Moisture (%)	Acidity (Degrees)	Water Activity (a_w_)
P0	12.48 ± 0.24 ^a^	1.67 ± 0.04 ^a^	0.524 ± 0.004 ^a^
P5	12.44 ± 0.17 ^a^	1.98 ± 0.02 ^b^	0.501 ± 0.005 ^ab^
P10	12.37 ± 0.31 ^a^	2.24 ± 0.03 ^c^	0.479 ± 0.006 ^b^
P15	12.05 ± 0.21 ^ab^	2.36 ± 0.06 ^cd^	0.465 ± 0.003 ^bc^
P20	11.76 ± 0.29 ^ab^	2.54 ± 0.05 ^d^	0.453 ± 0.005 ^c^
P30	11.62 ± 0.23 ^ab^	2.63 ± 0.02 ^de^	0.432 ± 0.002 ^d^
P40	11.36 ± 0.28 ^b^	2.71 ± 0.03 ^e^	0.428 ± 0.006 ^de^

Results indicate the mean value of three independent assays and are expressed as mean ± standard deviation (SD); in each column, different letters ^a–e^ mean significant differences (*p* ≤ 0.05). P0—control sample, P5—pretzel with 5% addition of hempseed cake, P10—pretzel with 10% addition of hempseed cake, P15—pretzel with 15% addition of hempseed cake, P20—pretzel with 20% addition of hempseed cake, P30—pretzel with 30% addition of hempseed cake, P40—pretzel with 40% addition of hempseed cake.

**Table 4:** Proximate composition of pretzels enriched with hemp seed cake.

Pretzels Sample	Protein (%)	Fat (%)	Fiber (%)	Ash (%)	Carbohydrate (%)	Energy (kcal/100 g)	Energy from Protein (%)
P0	7.49 ± 0.37 ^a^	3.13 ± 0.16 ^a^	0.90 ± 0.04 ^a^	1.79 ± 0.09 ^a^	74.20 ± 3.71 ^a^	354.93 ± 3.24 ^c^	8.44 ± 0.35 ^a^
P5	8.13 ± 0.41 ^b^	3.36 ± 0.17 ^ab^	2.26 ± 0.11 ^b^	1.98 ± 0.10 ^ab^	71.83 ± 3.59 ^ab^	350.08 ± 3.17 ^bc^	9.29 ± 0.42 ^ab^
P10	8.77 ± 0.44 ^bc^	3.59 ± 0.18 ^bc^	3.62 ± 0.18 ^c^	2.16 ± 0.11 ^b^	69.48 ± 3.47 ^b^	345.31 ± 2.45 ^b^	10.16 ± 0.15 ^b^
P15	9.41 ± 0.47 ^c^	3.83 ± 0.19 ^cd^	4.98 ± 0.25 ^d^	2.35 ± 0.12 ^bc^	67.38 ± 3.36 ^bc^	341.63 ± 3.05 ^b^	11.02 ± 0.53 ^b^
P20	10.05 ± 0.50 ^cd^	4.06 ± 0.20 ^de^	6.34 ± 0.32 ^e^	2.53 ± 0.13 ^cd^	65.25 ± 3.26 ^cd^	337.74 ± 2.11 ^ab^	11.90 ± 0.27 ^b^
P30	11.34 ± 0.57 ^d^	4.52 ± 0.23 ^ef^	9.06 ± 0.45 ^f^	2.90 ± 0.15 ^de^	60.56 ± 3.03 ^de^	328.28 ± 1.58 ^a^	13.82 ± 0.61 ^c^
P40	12.62 ± 0.63 ^e^	4.98 ± 0.25 ^f^	11.78 ± 0.59 ^g^	3.27 ± 0.16 ^e^	55.99 ± 2.80 ^e^	319.26 ± 2.45 ^a^	15.81 ± 0.41 ^d^

Results indicate the mean value of three independent assays and are expressed as mean ± standard deviation (SD); in each column, different letters ^a–g^ mean significant differences (*p* ≤ 0.05). P0—control sample, P5—pretzel with 5% addition of hempseed cake, P10—pretzel with 10% addition of hempseed cake, P15—pretzel with 15% addition of hempseed cake, P20—pretzel with 20% addition of hempseed cake, P30—pretzel with 30% addition of hempseed cake, P40—pretzel with 40% addition of hempseed cake.

**Table 5:** The bioactive value of pretzels enriched with hemp seed cake.

Pretzels Sample	Total Polyphenol Content (mg GAE/kg)	Total Flavonoid Content (mg GAE/kg)	DPPH (mg Trolox/kg)	ABTS (mg Trolox/kg)
P0	115.28 ± 2.14 ^a^	63.40 ± 1.21 ^a^	194.42 ± 2.75 ^a^	486.05 ± 6.9 ^a^
P5	193.54 ± 2.28 ^b^	107.45 ± 1.69 ^b^	285.75 ± 4.14 ^b^	714.38 ± 10.4 ^b^
P10	262.15 ± 1.64 ^c^	145.29 ± 1.48 ^c^	367.15 ± 3.35 ^c^	917.88 ± 8.4 ^c^
P15	314.93 ± 2.05 ^d^	175.40 ± 1.57 ^d^	453.46 ± 3.52 ^d^	1 133.65 ± 8.8 ^d^
P20	352,41 ± 1.78 ^e^	198.82 ± 1.42 ^e^	492.57 ± 4.21 ^e^	1 231.43 ± 10.5 ^e^
P30	412.71 ± 1.64 ^f^	232.47 ± 1.51 ^f^	576.34 ± 4.67 ^f^	1 440.85 ± 11.7 ^f^
P40	467.45 ± 2.11 ^g^	262.28 ± 1.68 ^g^	658.89 ± 5.34 ^g^	1 647.23 ± 13.3 ^g^

Results indicate the mean value of three independent assays and are expressed as mean ± standard deviation (SD); in each column, different letters ^a–g^ mean significant differences (*p* ≤ 0.05). P0—control sample, P5—pretzel with 5% addition of hempseed cake, P10—pretzel with 10% addition of hempseed cake, P15—pretzel with 15% addition of hempseed cake, P20—pretzel with 20% addition of hempseed cake, P30—pretzel with 30% addition of hempseed cake, P40—pretzel with 40% addition of hempseed cake.

**Table 6:** View of pretzel dough and final baked products across different formulations.

** 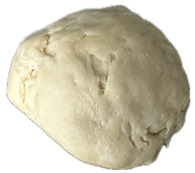 **	** 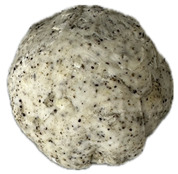 **	** 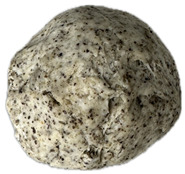 **	** 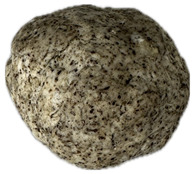 **
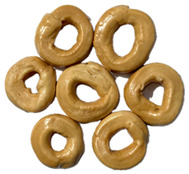	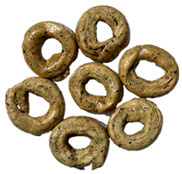	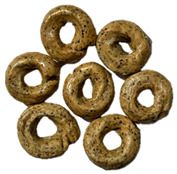	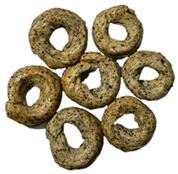
P0	P5	P10	P15
	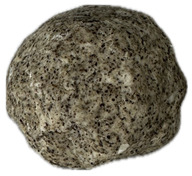	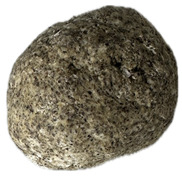	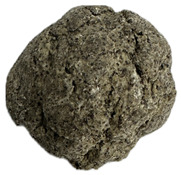
	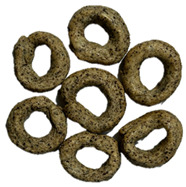	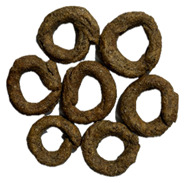	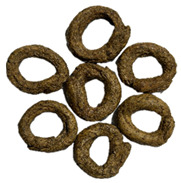
	P20	P30	P40

P0—control sample, P5—pretzel with 5% addition of hempseed cake, P10—pretzel with 10% addition of hempseed cake, P15—pretzel with 15% addition of hempseed cake, P20—pretzel with 20% addition of hempseed cake, P30—pretzel with 30% addition of hempseed cake, P40—pretzel with 40% addition of hempseed cake.

**Table 7:** Chromatic parameters of pretzel dough and final baked products.

Pretzels Sample	P0	P5	P10	P15	P20	P30	P40
Pretzel dough							
L*	97.40 ± 1.27 ^e^	90.25 ± 0.58 ^de^	87.06 ± 0.83 ^d^	77.35 ± 0.48 ^c^	62.34 ± 0.52 ^b^	59.23 ± 0.75 ^ab^	54.21 ± 0.59 ^a^
a*	−1.98 ± 0.04 ^b^	−2.42 ± 0.01 ^a^	−0.94 ± 0.02 ^c^	3.56 ± 0.02 ^g^	0.36 ± 0.01 ^f^	−0.12 ± 0.01 ^d^	0.19 ± 0.01 ^e^
b*	17.16 ± 0.31 ^c^	12.51 ± 0.23 ^a^	14.74 ± 0.45 ^b^	21.66 ± 0.68 ^d^	12.24 ± 0.29 ^a^	16.79 ± 0.34 ^bc^	12.77 ± 0.28 ^a^
Δ*E*	-	8.54 ± 0.11 ^a^	10.67 ± 0.14 ^b^	21.28 ± 0.21 ^c^	35.48 ± 0.54 ^d^	38.21 ± 0.29 ^e^	43.46 ± 0.63 ^f^
*WI*	82.53 ± 0.98 ^ef^	83.95 ± 0.85 ^f^	80.36 ± 1.02 ^e^	68.45 ± 0.75 ^d^	60.39 ± 0.67 ^c^	55.90 ± 0.68 ^b^	52.46 ± 0.46 ^a^
Final baked products							
L*	82.39 ± 0.95 ^f^	70.59 ± 0.85 ^e^	63.64 ± 0.86 ^de^	57.81 ± 0.65 ^d^	47.82 ± 0.25 ^c^	35.67 ± 0.25 ^b^	27.15 ± 0.32 ^a^
a*	3.08 ± 0.04 ^a^	5.84 ± 0.02 ^b^	3.64 ± 0.02 ^ab^	6.3 ± 0.05 ^c^	2.91 ± 0.01 ^a^	8.99 ± 0.11 ^d^	6.39 ± 0.35 ^c^
b*	27.64 ± 0.21 ^b^	29.98 ± 0.34 ^c^	26.90 ± 0.31 ^ab^	36.46 ± 0.45 ^d^	25.53 ± 0.28 ^a^	29.31 ± 0.65 ^c^	29.62 ± 0.54 ^c^
Δ*E*	-	12.34 ± 0.16 ^a^	18.77 ± 0.09 ^b^	26.31 ± 0.21 ^c^	34.63 ± 0.33 ^d^	47.12 ± 0.58 ^e^	55.37 ± 0.61 ^f^
*WI*	67.08 ± 0.84 ^g^	57.59 ± 0.78 ^f^	54.62 ± 0.65 ^e^	43.88 ± 0.38 ^d^	41.83 ± 0.58 ^c^	28.73 ± 0.32 ^b^	21.09 ± 0.17 ^a^

Results indicate the mean value of three independent assays and are expressed as mean ± standard deviation (SD); in each row, different letters ^a–g^ mean significant differences (*p* ≤ 0.05). P0—control sample, P5—pretzel with 5% addition of hempseed cake, P10—pretzel with 10% addition of hempseed cake, P15—pretzel with 15% addition of hempseed cake, P20—pretzel with 20% addition of hempseed cake, P30—pretzel with 30% addition of hempseed cake, P40—pretzel with 40% addition of hempseed cake.

## Data Availability

The original contributions presented in the study are included in the article, further inquiries can be directed to the corresponding author.
